# Esketamine improves postoperative sleep quality in thoracic surgery patients with preoperative sleep disturbance: a randomized controlled trial

**DOI:** 10.3389/fphar.2026.1843977

**Published:** 2026-06-23

**Authors:** Xingyu Wang, Haijin Wang, Huiyu Luo

**Affiliations:** Xiangyang NO.1 People’s Hospital, Hubei University of Medicine, Xiangyang, China

**Keywords:** esketamine, NMDA receptor antagonist, perioperative care, perioperative sleep disturbance, thoracic surgery

## Abstract

**Objective:**

To investigate the effect of multiple intraoperative subanesthetic doses of esketamine on postoperative sleep quality in non-cardiac thoracic surgery patients with preoperative sleep disturbance.

**Methods:**

This was a prospective single-center, double-blind placebo-randomized controlled trial of 100 patients undergoing elective non-cardiac thoracic surgery under general anesthesia with preoperative sleep disturbance who were given 0.25 mg/kg esketamine at induction of anesthesia and cutaneous suture compared with placebo to prevent the occurrence of postoperative sleep disturbance. Primary outcomes were the incidence of postoperative sleep disturbance on postoperative day 1, as assessed by Numeric Rating Scale, Athens Insomnia Scale and RCSQ. Secondary outcomes included the incidence of postoperative sleep disturbance on postoperative day 3 and 5,sleep duration, proportion of deep sleep, proportion of rapid eye movement sleep, postoperative pain score, postoperative anxiety score and depression score, intraoperative hemodynamic parameters and blood biomarkers (IL-6,IL-10, and brain-derived neurotrophic factor).

**Result:**

A total of 100 patients were randomized to the control group and the esketamine group. The incidence of postoperative sleep disturbance in the esketamine group was significantly lower than in the control group on POD 1 (50%vs 82%; odds ratio [OR], 0.2 [95% CI, 0.1–0.5]; P = 0.001) and POD 3 (22%vs 42%; OR, 0.4 [95% CI, 0.2–0.9]; P = 0.032). And there were significant differences in sleep duration, proportion of deep sleep and proportion of REM sleep (Based on exploratory Fitbit-derived estimates) on postoperative day 1 and 3. By POD 5, the difference was no longer statistically significant. Postoperative pain scores at rest and during movement were significantly lower in the esketamine group immediately after surgery and on postoperative day 1 compared with the control group, with no significant differences on postoperative days 3 and 5. Anxiety and depression scores (HADS) were significantly lower in the esketamine group on postoperative days 1, 3, and 5. Intraoperative hemodynamic parameters demonstrated higher systolic and mean arterial pressures in the esketamine group during anesthesia induction (T2) and lower pressures and heart rate during extubation (T6), indicating attenuated hypotension at induction and blunted stress response at extubation. Intraoperative sufentanil consumption was significantly lower in the esketamine group. Compared with preoperative levels, serum IL-6 concentrations increased in both groups on POD 1, 3, and 5; however, the esketamine group exhibited lower IL-6 levels than the control group on POD one and POD 3, with the difference attenuating by POD 5. Serum IL-10 levels were elevated on POD 1, 3, and five in the esketamine group and were significantly higher than those in the control group at all three time points. Serum BDNF concentrations were increased relative to baseline on POD 1, 3, and five in both groups, and were consistently higher in the esketamine group than in the control group across all postoperative assessments. Subgroup analyses demonstrated that intraoperative esketamine consistently reduced the incidence of postoperative sleep disturbance across predefined subgroups stratified by age, sex, PSQI score, and ASA classification, with no significant interaction effects observed.

**Conclusion:**

Multiple intraoperative infusion of 0.25 mg/kg esketamine can effectively improve the postoperative sleep quality of non-cardiac thoracic surgery patients with preoperative sleep disturbance, reduce the incidence of postoperative sleep disturbance, reduce the use of opioids, reduce the occurrence of intraoperative hypotension events, reduce the stress response during extubation, maintain the stability of intraoperative hemodynamics, reduce postoperative pain, and relieve adverse emotions. Moreover, esketamine decreased the level of the pro-inflammatory cytokine IL-6, increased the level of the anti-inflammatory cytokine IL-10, attenuated the postoperative inflammatory response, and promoted the release of BDNF.

**Clinical Trial Registration:**

https://www.chictr.org.cn/bin/project/edit?pid=248681, identifier ChiCTR2500096036.

## 1 Introduction

Sleep is a universal and fundamental biological process, defined as a naturally reversible state accompanied by loss of consciousness, reduced responsiveness to external stimuli and relative inactivity (Tononi et al., 2024; Rasch and Born, 2013). Sleep is divided into rapid eye movement (REM), and non-REM (Huang H. et al., 2021), which is subdivided into three stages: N1 (light sleep), N2 (moderate sleep), and N3 (deep sleep). Good sleep has positive effects on memory, cognition, immunity, mental health, cardiovascular health, reproductive health, and hormonal regulation (Baranwal et al., 2023). A sleep disorder is a clinical syndrome characterized by various disturbances in sleep-wake rhythm and abnormal sleep quality (Morin et al., 2020). It is common in perioperative patients, especially elderly patients undergoing major surgery (Dua et al., 2020). It is mainly manifested as difficulty falling asleep, sleep disruption and daytime fatigue (Zhang et al., 2023). Postoperative sleep disturbance not only affects patient recovery and prognosis, but also increase the risk of postoperative delirium and postoperative cognitive impairment (Wang et al., 2021). A high preoperative Pittsburgh Sleep Quality Index (PSQI) score, indicative of preoperative sleep disturbance, is a primary risk factor for postoperative sleep disturbance (Butris et al., 2023). Therefore, selecting patients with preoperative sleep disturbance as the intervention target can facilitate a better understanding of the impact of interventions on postoperative sleep disturbance. Unfortunately, however, there has been little research on the prevention of postoperative sleep disturbance.

Esketamine is the S-enantiomer of the NMDA antagonist ketamine, which has greater affinity, stronger analgesic and sedative effect, and fewer adverse reactions (Swainson et al., 2019). In clinical work, esketamine can be used alone for short operations, diagnostic operations, trauma first aid and other scenarios, and can also be combined with general anesthesia or regional anesthesia to provide sedation, analgesia, anesthesia. At the subanesthetic dose of esketamine(0.25 mg/kg), it not only shows a good therapeutic effect but also reduces the incidence of side effects associated with the dosage. Moreover, it demonstrates a strong antidepressant effect for patients with refractory depression. (Alnefeesi et al., 2022). Moreover, subanesthetic doses of esketamine have been shown to have definitive neuroprotective effects (Jumaili et al., 2022). In addition, esketamine has shown some potential for ameliorating perioperative sleep disorders, which may be related to its antidepressant efficacy, anti-inflammatory properties, analgesic efficacy, interaction with the circadian system, and neurocognitive and antianxiety effects (Song and Zhu, 2021). A randomized clinical trial by Qiu and colleagues showed that intraoperative infusion of esketamine reduced the incidence of postoperative sleep disorders in patients undergoing gynecological laparoscopic surgery (Qiu et al., 2022).

Preoperative PSQI score is high, that is, preoperative sleep disturbance is the main risk factor for postoperative sleep disturbance (Butris et al., 2023). Thoracic surgery is a common major procedure in surgical operations. About 79% of patients have sleep problems before surgery, and postoperative sleep disorders are often induced by reasons such as greater trauma and intense postoperative pain (Ida et al., 2019). Therefore, this study evaluated the effect of esketamine on postoperative sleep quality in thoracic surgery patients with preoperative sleep disturbance, and explored its potential mechanisms.

## 2 Methods and materials

### 2.1 Study design and ethics

This was a single-center, double-blind, prospective randomized controlled clinical trial. The ethical approval of this study was granted by the Ethics Committee of Xiangyang No.1 People’s Hospital affiliated to Hubei University of Medicine (identification number: 2024KY047). The study has been registered in the Chinese Clinical Trials Registry (ChiCTR2500096036). The work has been reported in line with CONSORT guidelines.

### 2.2 Participants

Patients in the trial were 18–80 years of age, had the American Society of Anesthesiologists (ASA) body status classification I to III (I indicating healthy patients, II indicating mild systemic disease, III indicating severe systemic disease), and were undergoing non-cardiac thoracic surgery at the Xiangyang No.1 People’s Hospital. The exclusion criteria are as follows: (1) The patient refused to participate in the study, (2) body mass index (measured as body weight in kilograms divided by height in meters squared) greater than 30, (3) preoperative PSQI less than 7, (4) recent history of substance abuse, (5) contraindications or allergies to eschlorotenone, (6) cognitive impairment or inability to communicate, (7) inability to use patient-controlled intravenous analgesia pumps, and (8) recent use (within 2 weeks) of medications known to affect sleep, such as benzodiazepines, non-benzodiazepine sedative-hypnotics (zolpidem, zopiclone), antidepressants with sedating properties, antipsychotics, or exogenous melatonin. The study was prospectively registered in the Chinese Clinical Trial Registry (ChiCTR2500096036; http://www.chictr.org.cn/) prior to patient enrollment.

### 2.3 Randomization and blinding

Eligible patients were evaluated the day before surgery. Using a computer-generated randomization table, the patients were randomly assigned to two groups at a ratio of 1:1, the experimental group received 0.25 mg/kg esketamine(2mL, 50mg, Hengrui Induction, Jiangsu,China) infusion during anesthesia induction and skin suture, and the control group received the same amount of normal saline. For covert dispensing, esketamine and saline were prepared to 20 mL in the same 20 mL syringe by a nurse who was not involved in any treatment or perioperative assessment. The anesthesiologists involved in patient management and postoperative follow-up were unaware of the group assignment. Group assignments were not announced until after patients were discharged from the hospital.

### 2.4 Anesthesia and postoperative analgesia management

Routine intraoperative monitoring was established, including invasive radial arterial blood pressure, electrocardiogram, carbon dioxide monitoring and pulse oxygen saturation. Anesthesia was induced by intravenous injection of remazolam (0.2 mg/kg), etomidate (0.3 mg/kg), sufentanil (The initial dose of sufentanil 0.4–0.6 μg/kg was titrated by the attending anesthesiologist based on patient age, body weight, comorbidities, and pre-induction hemodynamic status, with the goal of blunting the hemodynamic response to tracheal intubation. Intraoperative opioid administration was adjusted to maintain heart rate and blood pressure within 20% of baseline values.) and cis-atracurium (0.2 mg/kg). A double-lumen tracheal catheter was used for pulmonary isolation, and sevoflurane (1.5%–2%) and remifentanil (6–12 μg kg/h) were pumped continuously during the operation to maintain anesthesia. Neuromuscular block is maintained by intermittent injections of cis-atracurium as needed. Auscultation and fibroscopy were used to confirm lung isolation, and all patients received the same protective single-lung ventilation strategy (tidal volume 4–6 mL/kg, PEEP five cmH20, respiratory rate 12 to 15/min, and, ETCO2 maintenance between 35 and 45 cmH20). The fluctuation range of HR and BP was maintained within 20% of the basic value during the operation. The experimental group was given 0.25 mg/kg esketamine at the time of anesthesia induction and cutaneous suture, while the control group was given the same amount of normal saline. An intercostal nerve block was performed for analgesia before the end of the procedure, and after the procedure was completed, the patient was transferred to the post-anesthesia care unit for recovery. Residual muscle relaxants can be reversed with atropine and neostigmine if necessary. When the patient’s Alderete score was >9, the patient was allowed to be transferred to the ward. Routine patient-controlled intravenous analgesia was administered in all groups for the first 48 h after surgery, with hydromorphone (10 mg) and ondansetron (8 mg) dissolved in 100 mL normal saline. The pump offers a base infusion of 2 mL/h and a rapid infusion (0.5 mL, a 15 min lock-out time).

### 2.5 Outcome measurements

Preoperative sleep quality was assessed by Pittsburgh Sleep Quality Index (PSQI), Numeric Rating Scale (NRS), Athens Insomnia Scale (AIS) and Richards-Campbell Sleep Scale (RCSQ). Postoperative sleep quality was assessed by NRS, AIS and RCSQ. Sleep duration, proportion of deep sleep, and proportion of REM were monitored using a sleep monitoring bracelet (Fitbit) on preoperative day 1 and on postoperative day 1,3 and 5. Postoperative pain intensity was evaluated at rest and during movement (coughing/deep breathing) by a trained investigator who was blinded to group allocation, using an 11-point (0–10) visual analogue scale (VAS), where 0 represents no pain and 10 represents the worst imaginable pain. All patients received standardized patient-controlled intravenous analgesia (PCIA) as described, ensuring a consistent background analgesic regimen across both groups. Anxiety and depressive symptoms were assessed on preoperative day 1 and on postoperative days 1, 3, and five using the Hospital Anxiety and Depression Scale (HADS; Zigmond and Snaith, 1983), which yields separate subscale scores for anxiety (HADS-A) and depression (HADS-D), each ranging from 0 to 21.

The primary outcome were the incidence of postoperative sleep disturbance (PSD), which were assessed using NRS, AIS and RCSQ on postoperative day 1. Postoperative sleep disturbance was defined as the fulfillment of all three of the following criteria simultaneously: (1) an NRS score ≥6, (2) an AIS score ≥6, and (3) an RCSQ score ≤50, indicating that sleep was repeatedly disrupted throughout the night, or worse (Cai et al., 2020; Tonna et al., 2021). The secondary outcomes included hemodynamic parameters at T1 (5 min after entering the operating room), T2 (anesthesia induction), T3 (intubation), T4 (10 min after intubation), T5 (skin suture), T6 (extubation), and T7 (out of the operating room). Pain scores at rest and on movement at the end of surgery and on postoperative day 1, 3 and 5 (using VAS), anxiety and depression scores on postoperative day 1, 3 and 5 (using HADS), and hydromorphone intake on postoperative days 1 and 2. Other study parameters included intraoperative use of pressors, intraoperative consumption of opioids, intraoperative use of neostigmine, extubation time, infusion, blood loss, urine volume, adverse reactions (nausea and vomiting, dizziness, itching, and nightmares), pulmonary complications, surgery duration, anesthesia duration, duration of chest tube retention, and duration of hospital stay after surgery.

Blood samples were collected on preoperative day 1 and on postoperative day 1,3 and 5, and then stored in a 4 °C refrigerator. These samples were centrifuged at 3,000 rpm for 10 min to isolate the serum, which was stored at −80° C for further testing. Serum concentrations of pro-inflammatory factors (interleukin-6, IL-6), anti-inflammatory factors (interleukin-10, IL-10), and brain-derived neurotrophic factor (BDNF) were assessed by enzyme-linked immunosorbent assay (ELISA).

### 2.6 Statistical analysis

According to the literature and pre-experimental results (Baranwal et al., 2023; Ida et al., 2019), it is estimated that the incidence of postoperative sleep disorders in patients with preoperative sleep disorders is about 80%. After the application of esketamine, the incidence of postoperative sleep disorders is reduced to 50%, bilateral α = 0.05, and power is 80%, a total of 78 patients are required for this study. Accounted for an estimated 10% attrition rate, 100 patients were included in the trial.

Statistical analysis was performed using SPSS, version 25.0 (IBM SPSS). Baseline characteristics between the groups were compared using standardized differences, defined as absolute differences in means or proportions divided by the pooled standard deviation. Baseline characteristics were considered unequal between groups with absolute standardized differences exceeding 1.96 × $\sqrt{\left[n1+n2\right]÷\left[n1\times n2\right]}$ = 0.392. The normal distribution of the variables was examined using the Kolmogorov-Smirnov test. Continuous data were expressed as mean ± standard deviation, compared using independent sample T-test if they were normally distributed. And data that were non normal distribution was reported as median (quartile) and analyzed using Mann-Whitney test. For non-normally distributed variables analyzed with the Mann-Whitney U test, the median difference between groups and its 95% confidence interval were estimated using the Hodges-Lehmann method. The generalized estimating equation (GEE) model was used to evaluate the impact of repeated measurement data and research factors on the research objective. All models were adjusted for pre-defined latent covariates, including age, gender, ASA classification, smoking status, drinking status, type of surgery, underlying disease, duration of surgery and duration of anesthesia, Bonferroni correction was used to adjust the thtreshold for tests at multiple time-points (p < 0.005). Categorical variables were reported numerically (%), and compared using χ2 or Fisher exact tests, as appropriate. Correlations between levels of IL-6, IL-10, and BDNF on postoperative day 1 and sleep parameters (assessed by the RCSQ) were assessed using Spearman’s rank correlation analysis. Prespecified subgroup analyses (age,sex,ASA physical status classification, and PSQI point) were performed using logistic regression models to calculate the treatment -by-covariate interactions. Time-to-event datawere evaluated with Kaplan-Meier survival analysis, with between group difference testedwith the log-rank test. Considering multiplicity, we adopt a strategy of hierarchical testing. The authors considered that p < 0.05 (double-tailed) was statistically significant.

## 3 Results

### 3.1 Demographic and clinical characteristics of patients

**FIGURE 1:**
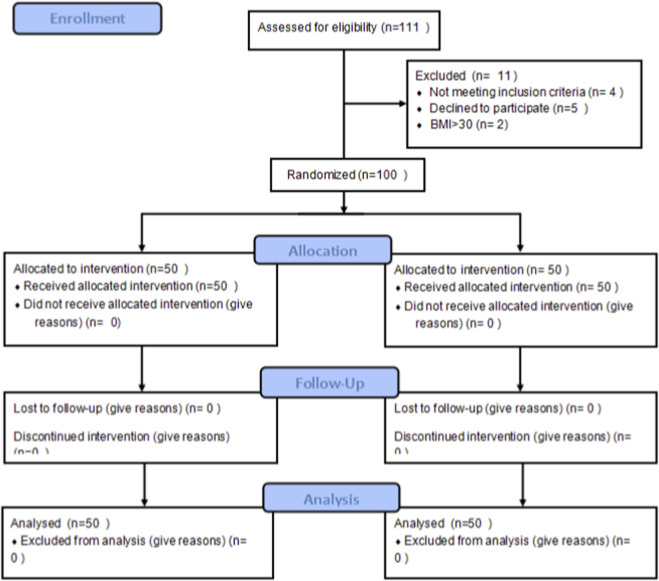
CONSORT flow diagram of participant enrollment and randomization.

Out of the 111 eligible patients screened during the study, six were excluded based on exclusion criteria and 5 declined to participate (Figure 1). Therefore, 100 patients were recruited and randomly assigned to one of two groups. Demographic data such as age, sex, BMI, ASA physical status classification, education level, smoking status and alcohol consumption status were not significantly different between the two groups (Table 1). We collected and compared the two groups of patients with preoperative, intraoperative and postoperative clinical characteristics and outcomes, which including intraoperative vasopressor use, intraoperative neostigmine use, extubation time, fluids infusion, blood loss, urine, adverse events (nausea and vomiting, dizziness, pruritis and nightmares), pulmonary complications, operation duration, anesthesia duration, consumption of hydromorphone 24 and 48 h after surgery, chest tube retention time, and length of hospital stay after surgery (Table 2).

**TABLE 1:** Patient demographic data.

Variable	Control (n = 50)	Experimental (n = 50)	P value
Age (y)	65.88 ± 5.03	65.92 ± 4.27	0.09
BMI (kg/m^2^)	23.06 ± 3.71	23.48 ± 3.98	0.109
Sex, n (%)	​	​	0.040
Male	26 (52)	25 (50)	​
Female	24 (48)	25 (50)	​
ASA, n (%)	​	​	0.051
I	3 (6)	2 (4)	​
II	37 (74)	39 (78)	​
III	10 (20)	9 (18)	​
Smoking status, n (%)	​	​	0.059
Current smoker	23 (46)	24 (48)	​
Former smoker	21 (42)	19 (38)	​
Nonsmoker	6 (12)	7 (14)	​
Drinking status, n (%)	​	​	0.085
Current drinker	31 (62)	28 (56)	​
Former drinker	9 (18)	10 (20)	​
Nondrinker	10 (20)	12 (24)	​

Standardized differences >0.392 are considered as inequality between two groups.

**TABLE 2:** Sleep related indicators.

Time point / indicator	Control	Experimental	Difference / risk ratio / hazard ratio	P value
​	n = 50	n = 50	95%CI	​
Preoperative day 1				
PSQI score	12 (10,15)	12 (10,15)	-	0.822
AIS score	6 (5,8)	6 (6,8)	-	0.484
NRS score	2 (0.5,3)	2 (0,3.5)	-	0.835
RCSQ score	64 (62,66)	64 (62,66)	-	0.502
Sleep duration (h)	6.20 ± 0.98	6.20 ± 1.09	-	0.993
Deep sleep proportion (%)	17.55 ± 1.32	17.66 ± 1.55	-	0.703
REM sleep proportion (%)	17.74 ± 2.07	17.96 ± 2.05	-	0.594
Postoperative day 1				
PSD (%)	41 (82)	25 (50)	0.2 (0.1–0.5)	0.001
AIS score	6 (6,9)	5 (5,9)	1 (0–2)	0.029
NRS score	3 (3,4)	3 (2.75,3.25)	0 (0–0)	0.025
RCSQ score	53 (52,54)	54 (53,56)	−1 (-2 to −1)	<0.001
Sleep duration (h)	5.45 ± 0.90	5.92 ± 1.03	−0.5 (-0.8 to −0.1)	0.018
Deep sleep proportion (%)	15.01 ± 1.03	15.59 ± 1.28	−0.6 (-1 to −0.1)	0.015
REM sleep proportion (%)	16.22 ± 1.91	17.06 ± 1.74	−0.8 (-1.6 to −0.1)	0.024
Postoperative day 3				
PSD (%)	21 (42)	11 (22)	0.4 (0.2–0.9)	0.032
AIS score	5 (5,6)	5 (4,5)	1 (0–1)	0.029
NRS score	2 (1,2)	2 (1.2)	-	0.235
RCSQ score	57 (55,58)	57.5 (57,59)	−1 (-2,0)	0.020
Sleep duration (h)	5.85 ± 0.87	6.25 ± 0.94	−0.4 (-0.8 to 0)	0.032
Deep sleep proportion (%)	16.85 ± 1.34	17.46 ± 1.29	−0.6 (-1.1 to −0.1)	0.023
REM sleep proportion (%)	17.66 ± 1.84	18.14 ± 1.92	−0.5 (-1.2 to −0.3)	0.021
Postoperative day 5				
PSD (%)	9 (18)	5 (10)	​	0.251
AIS score	4 (4,5)	4 (4,5)	-	0.554
NRS score	1 (0,1)	1 (0,1)	-	0.582
RCSQ score	63 (62.75,65)	64 (63,65)	-	0.533
Sleep duration (h)	6.19 ± 0.84	6.35 ± 0.95	-	0.384
Deep sleep proportion (%)	17.67 ± 1.31	17.90 ± 1.25	-	0.384
REM sleep proportion (%)	18.30 ± 1.80	18.44 ± 1.72	-	0.692

Deep sleep proportion and REM, sleep proportion were derived from Fitbit-based exploratory estimates and should be interpreted with caution.

Abbreviations: AIS, athens insomnia scale; NRS, numeric rating scale; POD, postoperative day; PSD, postoperative sleep disturbance; PSQI, pittsburgh sleep quality index; RCSQ, Richards-Campbell Sleep Questionnaire; REM, rapid eye movement.

### 3.2 Sleep related indicators

There were no significant differences in PSQI scores, AIS scores, NRS scores, RSCQ scores, sleep duration, deep sleep proportion, and REM sleep proportion between the two groups before surgery. The incidence of postoperative sleep disturbance, during the initial five postoperative days was significant differences between the two groups (Figure 5). On postoperative day 1, the experimental group showed better outcomes in terms of sleep disturbance incidence, AIS scores, NRS scores, RSCQ scores, sleep duration, deep sleep proportion, and REM sleep proportion compared to the control group. On postoperative day 3, the experimental group continued to exhibit higher values in sleep disturbance incidence, AIS scores, RSCQ scores, sleep duration, deep sleep proportion, and REM sleep proportion than the control group, with no significant difference in NRS scores between the two groups. On postoperative day 5, there were no significant differences in sleep related indicators between the two groups (Table 2). Esketamine comparably reduced the incidence of postoperative sleep disturbance across various predefined subgroups,and no significant interactions were identified (Figure 6) (Regarding the duration of sleep, the results of deep sleep time and REM sleep time are based on the preliminary findings of the Fitbit estimation).

### 3.3 Clinical characteristics and outcomes

We collected and compared the two groups of patients with preoperative, intraoperative and postoperative clinical characteristics and outcomes, which including intraoperative vasopressor use, intraoperative neostigmine use, extubation time, fluids infusion, blood loss, urine, adverse events (nausea and vomiting, dizziness, pruritis and nightmares), pulmonary complications, operation duration, anesthesia duration, consumption of hydromorphone 24 and 48 h after surgery, chest tube retention time, and length of hospital stay after surgery (Table 3).

**TABLE 3:** Clinical characteristics and outcomes.

Variable	Control (n = 50)	Experimental (n = 50)
Surgical site,n (%)		
Left lung	19 (38)	22 (44)
Right lung	20 (40)	19 (38)
Mediastinum	11 (22)	9 (18)
Surgery type,n (%)		
Lobectomy	28 (56)	30 (60)
Segmentectomy	10 (20)	9 (18)
Wedge resection	2 (4)	3 (6)
Pulmonary lesions resection	1 (2)	0 (0)
Mediastinal mass resection	6 (12)	4 (8)
Mediastinal tumor resection	5 (10)	5 (10)
Disease,n (%)		
Diabetes	35 (70)	31 (62)
Hypertension	39 (78)	40 (80)
Heart disease	11 (22)	14 (28)
Cerebral infarction	6 (12)	7 (14)
Liver disease	7 (14)	8 (16)
Kidney disease	15 (30)	18 (36)
Vasopressor use, n (%)	24	26
Neostigmine use, n (%)	29	32
Consumption of intraoperative opioid		
Sufentanil (μg)	35 (30,40)	30 (25,40)*
Remifentanil (μg/kg/min)	0.15 (0.14,0.18)	0.15 (0.14,0.18)
Operation duration (min)	147.90 ± 41.58	151.40 ± 40.59
Anesthesia duration (min)	191.30 ± 50.11	193.50 ± 53.45
Extubation time (min)	20.50 ± 6.79	22.30 ± 5.94
Fluids infusion (mL)	1,000 (900,1100)	1,000 (900,1100)
Blood loss (mL)	50 (30,100)	50 (40,100)
Urine (mL)	500 (400,600)	500 (400,600)
VAS scores at rest		
Immediately after surgery	3 (3,3)	3 (2,3)*
Postoperative day 1	3.5 (3,4)	3 (3,3)*
Postoperative day 3	2 (2,2)	2 (1,2)
Postoperative day 5	1 (0,1)	1 (0,1)
VAS scores for movement	​	3 (3,3)
Immediately after surgery	3 (3,3)	3 (3,3)*
Postoperative day 1	4 (3,4)	3 (3.4)*
Postoperative day 3	2 (2,2)	2 (2,2)
Postoperative day 5	1 (0,1)	0 (0,1)
Postoperative adverse events,n (%)		
N ausea and vomiting	7 (14)	5 (10)
Dizziness	2 (4)	1 (2)
Pruritis	0	0
Nightmares	0	0
Delirium	5 (10)	7 (14)
Consumption of hydromorphone after surgery (mg)		
24 h after surgery	5 (4.5,5)	5 (4,5)
48 h after surgery	9 (8.5,10)	9 (8.5,9.5)
Pulmonary complication, n (%)	5 (10)	3 (6)
Chest tube retention time (d)	4 (3,5)	4 (3,5)
Length of hospital stay after surgery (d)	6.5 (6,7)	6 (5,7)

*p < 0.05 vs. Control group at the same time.

Abbreviations: AIS, athens insomnia scale; NRS, numeric rating scale; POD, postoperative day; PSD, postoperative sleep disturbance; PSQI, pittsburgh sleep quality index; RCSQ, Richards-Campbell Sleep Questionnaire; REM, rapid eye movement.

**FIGURE 2:**
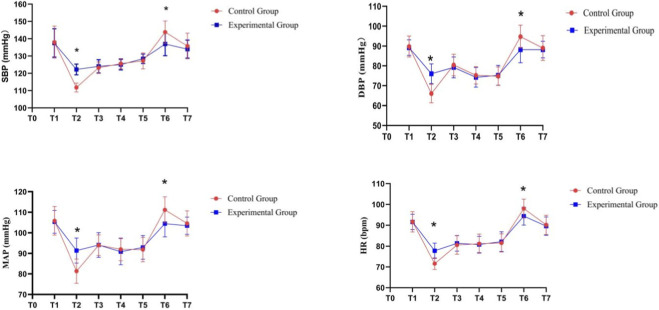
Hemodynamic parameters. Note: Data are presented as mean ± SD,*p < 0.005 vs. Control group at the same time.

At different time points, the comparison of SBP (systolic blood pressure), DBP (diastolic blood pressure), MAP (mean arterial pressure), and HR (heart rate) showed that the experimental group was higher than the control group at T2, and lower than the control group at T6, indicating that esketamine can reduce the occurrence of hypotension during anesthesia induction and can also reduce the stress response during extubation, maintaining hemodynamic stability. But the hemodynamic differences observed at T2 should be interpreted with caution, as they likely reflect a composite effect of two interrelated factors: the direct sympathomimetic action of esketamine that may counteract hypotension, and the reduced sufentanil dose in the esketamine group, which may have attenuated the opioid-mediated hypotensive effect. The relative contributions of these two mechanisms cannot be disentangled in the present study design. At other time points, there were no significant differences, and no events of increased heart rate or elevated blood pressure caused by esketamine were observed (Figure 2). The intraoperative infusion dose of sufentanil in the experimental group was lower than that in the control group (p = 0.044, median different 5.95%CI 0–5), while there was no significant difference in the intraoperative infusion dose of remifentanil. Additionally, the VAS scores for both movement (immediately post-operation: p = 0.025,median different 0.95%CI -1 to1; postoperative day 1: p = 0.037,median different 1, 95%CI -1–2) and rest (immediately post-operation: p = 0.023,median different 0.95%CI -1 to1; postoperative day 1: p = 0.01,median different 1, 95%CI -1to0) in the experimental group were lower than those in the control group immediately after surgery and on postoperative day 1, with no significant differences observed at other postoperative time points (Table 2).

### 3.4 Blood biomarkers and emotional performance

**FIGURE 3:**
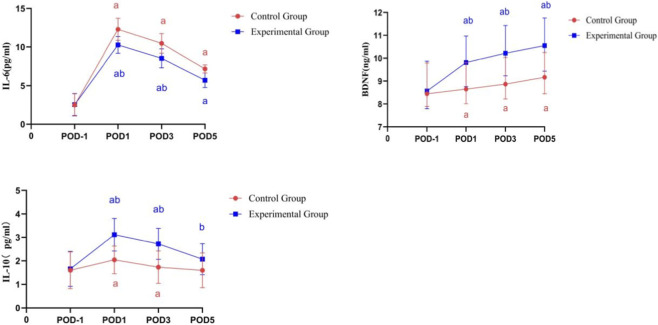
Blood biomarkers. Note: Data are presented as mean ± SD or median with IQR. ^a^p < 0.005:VS. Same group at POD -1 ^b^p < 0.005: VS. Control group at the same time. Abbreviations: BDNF, brain-derived neurotrophic factor; IL-6, interleukin-6; IL-10, interleukin-10.

There were no significant differences in IL-6, IL-10, and BDNF levels in the serum between the two groups before surgery. Compared to pre-surgery levels, serum IL-6 levels in both groups increased on postoperative day 1,3 and 5. However, the serum IL-6 levels in the experimental group were lower than those in the control group on postoperative day 1 and 3, with no significant difference between the two groups on postoperative day 5. Compared to pre-surgery levels, serum IL-10 levels in the control group increased on postoperative day 1 and 3, with no significant difference on postoperative day 5, in contrast, serum IL-10 levels in the experimental group increased on postoperative day 1,3 and 5, with significant differences between the experimental and control groups on all 3 days. Serum BDNF levels in both groups were higher on postoperative day 1,3 and 5 compared to pre-surgery levels, with significant differences between the two groups on all 3 days (Figure 3). Spearman correlation analysis indicated a strong negative correlation between postoperative day 1 serum IL-6 levels and RCSQ scores (r_s_ = −0.72, P < 0.001), a moderate positive correlation for IL-10 (r_s_ = 0.48, P = 0.007), and no significant correlation for BDNF (r_s_ = 0.18, P = 0.34).

**FIGURE 4:**
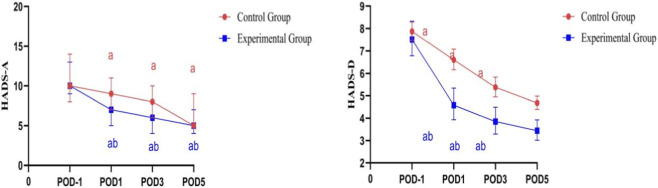
Emotional performance. Note: Data are presented as median with IQR. ^a^p < 0.005:VS. Same group at POD-1 ^b^p < 0.005: VS. Control group at the same time.

In terms of emotional expression, there were no significant differences in anxiety (HADS-A) and depression (HADS-D) scores between the two groups before surgery. Compared to pre-surgery, there were significant differences in both anxiety and depression scores in both groups. The anxiety scores of the experimental group were lower than those of the control group on postoperative day 1 and 3, but there was no significant difference on postoperative day 5. As for depression scores, the experimental group consistently scored lower than the control group (Figure 4).

### 3.5 Cumulative incidence and subgroup analysis

**FIGURE 5:**
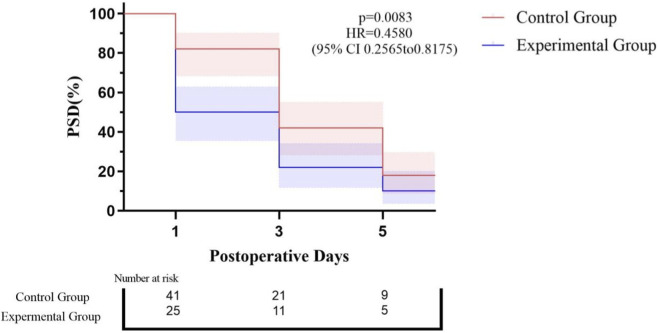
Cumulative incidence of postoperative sleep disturbance by two groups. A log-rank test was used to compare sleep disturbance distribution.

**FIGURE 6:**
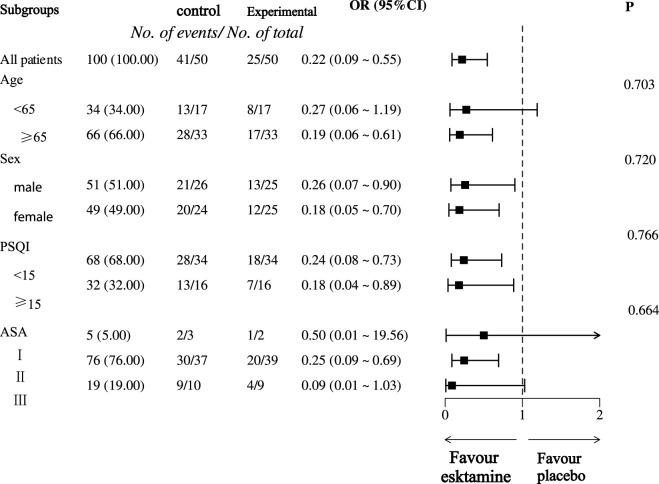
Risk ratios for postoperative sleep disturbance by subgroups.

The incidence of postoperative sleep disturbance, during the initial five postoperative days was significant differences between the two groups (Figure 5). Esketamine comparably reduced the incidence of postoperative sleep disturbance across various predefined subgroups,and no significant interactions were identified (Figure 6). Esketamine comparably reduced the incidence of postoperative sleep disturbance across all predefined subgroups, including those stratified by sex (P for interaction = 0.70), indicating that the beneficial effect was consistent in both male and female patients.

## 4 Discussion

In this randomized controlled trial, multiple intraoperative infusions of 0.25 mg/kg esketamine effectively improved postoperative sleep qualityof non-cardiac thoracic surgery patients with preoperative sleep disturbance, prolonged deep sleep and REM sleep, and reduced the incidence of sleep disturbance, which contributed to patients’ postoperative recovery. Additionally, esketamine decreased IL - 6, increased IL - 10, reduced the inflammatory response, and promoted the release of BDNF, which might be associated with the mechanism by which esketamine improves sleep. Similarly, esketamine reduced the use of opioids, lowered postoperative pain scores, and decreased the occurrence of negative emotions in patients, which also improved sleep. Moreover, esketamine reduced the incidence of intraoperative hypotensive events, mitigated the stress response during extubation, and maintained the stability of intraoperative hemodynamics.

Postoperative sleep disturbance (PSD) occurs in approximately 15%–72% of patients undergoing different types of surgeries, mainly manifesting as sleep deprivation, circadian rhythm disorder, and abnormal sleep structure (Su and Wang, 2018). PSD is influenced by a variety of complex factors, including patient age, surgical trauma, anesthesia method, postoperative pain, postoperative complications, anesthetic analgesics, environmental factors, and psychological factors (Luo et al., 2020). However, PSD not only increases the risks of delirium, cognitive impairment, and cardiovascular complications, enhances pain perception, prolongs hospital stay, and delays postoperative recovery but also has adverse effects on psychomotor function, emotional and metabolic functions, catabolic reactions, immunity, and inflammation (Hillman, 2017). Therefore, more in - depth research is needed for the diagnosis and treatment of PSD. This study demonstrates that even in patients at high risk of sleep disturbance, esketamine has a positive effect on postoperative sleep quality and can reduce the incidence of PSD.

The effects of general anesthetic drugs on sleep are dual-sided. On one hand, they can promote sleep. For example, a basic study showed that general anesthetic drugs can effectively restore the sleep behavior of sleep - deprived rats (Tung et al., 2004). Additionally, this study demonstrated the promoting effect of subanesthetic doses of esketamine on postoperative sleep quality. Moreover, a study reported that the α^2^ - adrenergic receptor agonist dexmedetomidine can increase N2 sleep, reduce sleep disturbances, and lower the incidence of postoperative sleep disorders, indicating that dexmedetomidine and esketamine have a synergistic effect in improving sleep quality (Chamadia et al., 2020; Huang X. et al., 2021). On the other hand, they can cause sleep problems. For instance, the inhalational anesthetics sevoflurane and isoflurane can disrupt the sleep structure and lead to sleep disturbances (Zhang et al., 2016). Therefore, we should learn to utilize the beneficial effects of anesthetic drugs on sleep to better improve patients’ postoperative sleep and reduce the incidence of sleep disorders. We also need to take into account the adverse effects of anesthetic drugs on sleep, reduce drug dosages, and implement personalized protocols to promote patients’ sleep and prognosis.

Esketamine, the dextrorotatory enantiomer of ketamine, demonstrates significant pulmonary protective effects by enhancing alveolar ventilation while preserving airway reflexes and respiratory drive. Furthermore, it relaxes bronchial smooth muscles, prevents bronchospasm, alleviates symptoms in patients with bronchospasm, and enhances pulmonary compliance. Our findings reveal that esketamine administration modulates inflammatory responses through suppressing pro-inflammatory IL-6 while elevating anti-inflammatory IL-10 levels. This immunomodulatory action attenuates systemic inflammation, potentially reducing acute lung injury incidence and pulmonary infection ri,promote postoperative pulmonary function exercise and respiratory recovery, decrease the incidence of atelectasis, and exert lung-protective effects. Given the short elimination half-life of esketamine (approximately 3–5 h), the sustained differences in serum BDNF and IL-10 concentrations observed on POD five cannot be solely attributed to the two intraoperative doses. Rather, these late-phase changes may reflect the initiation of a cascade of enduring neurobiological effects—including enhanced synaptic plasticity and sustained anti-inflammatory signaling—and/or may be influenced by postoperative confounders such as improved pain control, reduced opioid consumption, and ameliorated emotional distress. Notably, our study identified novel benefits of esketamine on sleep architecture restoration. While polysomnographic analysis demonstrated surgical patients (predominantly elderly with preoperative sleep disorders) typically exhibit reduced deep sleep (normally 20%–25% of total sleep time) and REM sleep (typically 25%) (Wei et al., 2023; Hillman, 2021), exploratory analyses based on Fitbit-derived estimates suggested that esketamine administration was associated with increased deep sleep and REM sleep duration postoperatively, these findings must be interpreted with considerable caution given the inherent limitations of consumer-grade wearable devices in accurately discriminating sleep stages. Definitive conclusions regarding esketamine’s effects on sleep architecture await confirmation in studies employing full polysomnography. Given the established correlation between sleep architecture preservation and enhanced wound healing, emotional regulation, and postoperative recovery, these findings suggest esketamine confers comprehensive perioperative advantages in thoracic surgery patients. Compared with other measures to improve perioperative sleep such as dexmedetomidine, esketamine has a rapid and powerful antidepressant effect and definite analgesic effect. This is particularly beneficial for patients who already have anxiety and depression before surgery and are prone to depressive mood after surgery. Moreover, it provides a unique multimodal path for improving perioperative sleep. Its value lies in simultaneously targeting the three closely interconnected areas of sleep, emotion and pain. However, the adverse mental reactions are also worthy of attention. However, it should be noted that recent studies have reported inconsistent effects of subanesthetic esketamine on postoperative anxiety and depressive symptoms (Song et al., 2025; Luo et al., 2024), suggesting that its psychological benefits may vary depending on surgical population, baseline emotional status, and the assessment instruments employed. In the present study, the observed improvement in HADS scores may be partially mediated by esketamine’s analgesic effects and its enhancement of sleep quality, rather than representing a direct, primary anxiolytic or antidepressant action.

The multifaceted mechanisms underlying esketamine’s effects on postoperative sleep quality may involve its antidepressant, anti-inflammatory, analgesic, and circadian regulatory properties. Regarding its antidepressant and mood-modulating properties, while esketamine is an NMDA receptor antagonist, its rapid and sustained emotional effects cannot be attributed solely to NMDA receptor blockade *per se*. Notably, other agents possessing acute NMDA antagonist properties—such as memantine, amantadine, dextromethorphan, or riluzole—do not consistently produce the rapid antidepressant responses characteristic of ketamine and esketamine. The unique effects of esketamine are thought to involve the inhibition of NMDA receptors located specifically on GABAergic interneurons, which disinhibits glutamatergic pyramidal neurons, leading to enhanced glutamate release, subsequent activation of postsynaptic AMPA receptors, and downstream stimulation of the mammalian target of rapamycin (mTOR) signaling cascade. This mTOR activation promotes the synthesis and release of brain-derived neurotrophic factor (BDNF), a molecular pathway consistent with the findings of the present study. BDNF has been associated with increased slow-wave sleep and improved sleep quality (Xue et al., 2023; Duncan et al., 2017). Froman analgesic and anti-inflammation perspective, surgical pain is the primary influencing factor of PSD. Pain signals can be transmitted to the brain through the spinal-thalamic-prefrontal pathway, thereby affecting sleep quality (Albinni et al., 2023; Lavigne et al., 2004). Esketamine exhibits potent analgesic effects and reduces the use of opioids, showing potential in improving sleep. Sleep disturbance can promote the upregulation of inflammatory factors such as IL-6. Similarly, inflammatory responses lead to sleep interruptions (Besedovsk et al., 2019). Esketamine reduces the release of the pro-inflammatory factor IL-6 and promotes the generation of the anti-inflammatory factor IL-10, thereby alleviating inflammatory responses. In terms of circadian rhythms, ketamine activates mTOR, and mTOR signaling is controlled by circadian rhythms, thereby regulating the transcription of circadian proteins. In addition, in the suprachiasmatic nucleus, mTOR is involved in the light-induced translation of core clock and clock-controlled genes, while the core clock protein BMAL1 is regulated by mTOR through its phosphorylation at the Ser42 site (Sato et al., 2022).

Some limitations of the study are worth noting: (1) This study is a single-center study, and future multi-center, large-sample studies are needed to further explore the effect of intraoperative infusion dose of esketamine on postoperative sleep quality in non-cardiothoracic surgery patients with sleep disorders before surgery; (2) This study only observed the sleep quality of patients in the short term after surgery, and the long-term sleep quality needs to be further studied; (3) Polysomnography is the gold standard of sleep monitoring, and this study did not use polysomnography for monitoring; (4) Due to the limited conditions, the related factors affecting sleep such as ward environment and night nursing are difficult to control, which may affect the accuracy of the study results. (5) Bispectral index (BIS) monitoring was not employed to assess depth of anesthesia in this study due to equipment availability constraints. Although esketamine has been reported to affect BIS readings, the absence of objective hypnotic depth monitoring may introduce potential confounding, as between-group differences in anesthetic depth could influence the observed outcomes. (6) Esketamine has a short half-life. The changes in biochemical indicators on the fifth day after surgery cannot be solely attributed to the intraoperative intervention. Future studies need to consider the influence of postoperative factors. Future studies with larger sample sizes may consider more granular stratified analyses—for example, by menopausal status in women or by more refined age categories—to explore whether subtle differential responses exist that were not detectable in the present subgroup analyses. Additionally, incorporating hormonal biomarkers (e.g., estradiol, progesterone) could provide mechanistic insight into potential sex-dependent effects.

## 5 Conclusion

In this study, we found that multiple intraoperative infusions of 0.25 mg/kg esketamine can effectively improve postoperative sleep quality in non-cardiac thoracic surgery patients with preoperative sleep disturbance, reduce the incidence of postoperative sleep disturbance, reduce the use of opioids, decrease the incidence of intraoperative hypotension, mitigate stress responses during extubation, maintain intraoperative hemodynamic stability, alleviate postoperative pain score, relieve negative emotions, and lower the levels of the pro-inflammatory cytokine IL-6 while increasing the levels of the anti-inflammatory cytokine IL-10, thereby reducing postoperative inflammatory responses, promote the release of BDNF.

## Data Availability

The raw data supporting the conclusions of this article will be made available by the authors, without undue reservation.
